# Risk factors for invasive mold infection after COVID-19: case-control study

**DOI:** 10.4314/ahs.v24i4.10

**Published:** 2024-12

**Authors:** Esma Eryilmaz-Eren, Hafize Sav, Zuhal Ozer-Simsek, İbrahim Ozcan, Aysin Kilinc-Toker, Azade Kanat, Ali Cetinkaya, Recep Civan Yuksel, Kaniye Aydin, Seda Guzeldag, Ilhami Celik

**Affiliations:** 1 Kayseri City Training and Research Hospital, Department of Infectious Diseases and Clinical Microbiology, Kayseri, Turkey; 2 Kayseri City Training and Research Hospital, Department of Medical Mycology, Kayseri, Turkey; 3 Kayseri City Training and Research Hospital, Department of Intensive Care, Kayseri, Turkey; 4 Kayseri City Training and Research Hospital, Department of Otorhinolaryngology-Head and Neck Surgery, Kayseri, Turkey; 5 Kayseri City Training and Research Hospital, Department of Infectious Disease and Clinical Microbiology, Kayseri, Turkey; 6 University of Health Sciences, Kayseri City Education and Research Hospital, Department of Infectious Disease and Clinical Microbiology, Kayseri, Turkey; 7 Kayseri City Training and Research Hospital, Department of Intensive Care, Kayseri, Turkey; 8 Erciyes University, Faculty of Medicine, Department of Intensive Care, Kayseri, Turkey; 9 Cukurova University, Faculty of Medicine, Department of Intensive Care, Adana, Turkey; 10 Seyhan State Hospital, Department of Intensive Care, Adana, Turkey

**Keywords:** Invasive mold infections, COVID-19, mucormycosis, invasive fungal sinusitis, invasive pulmonary aspergillosis

## Abstract

**Background:**

Invasive mold infections (IMI) have become common in patients with severe COVID-19 pneumonia, which are difficult to diagnose and treat, with a high mortality rate.

**Objective:**

The aim of this study was to determine risk factors for invasive mold infections associated with COVID-19.

**Methods:**

In this prospective, case-control study, patients treated for severe COVID-19 pneumonia in intensive care units with invasive mold infection were compared with severe COVID-19 pneumonia patients with no secondary infection (bacterial or fungal). Demographics, treatments received and outcomes were compared.

**Results:**

Twenty patients were included in the IMI group and 19 patients in the control group. Invasive aspergillosis was observed in 13 patients (65.0%) while mucormycosis was observed in seven patients (35.0%). Demographics and clinical characteristics were similar between IMI and control group (p>0.005). The 28-day mortality was 60.0% in the IMI group and 15.8% in the control group (p=0.005). The use of steroids has been identified as the most important risk factor for developing IMI (90.0% vs. 15.8%, OR: 25.712, p=0.009).

**Conclusion:**

Rationale use of steroids, with appropriate indication, dose and duration is important in the treatment of severe COVID-19 pneumonia.

## Introduction

The clinical manifestations of COVID-19 can range from asymptomatic to life-threatening respiratory failure.^1^ In severe or critical cases, increased levels of pro-inflammatory cytokines such as IL-6 and increased inflammation have been shown.^2^ Corticosteroids and immunomodulatory drugs are recommended and frequently used because they reduce mortality.^3^-^5^ Also, immunodeficiency is seen in severe COVID-19 cases. Lymphocyte damage and immunosuppression in the pathogenesis of COVID-19 predispose to secondary infections.^6^ On the other hand, invasive mold infections (IMIs) associated with COVID-19 are difficult to diagnose and treat, with a high mortality rate.^7^-^8^

It has been determined that COVID-19 facilitates the development of pulmonary aspergillosis. The definition of COVID-19-associated pulmonary aspergillosis (CAPA) has entered the literature.^9^ CAPA can develop both with damage to the alveolar and respiratory epithelium after COVID-19, and also due to immune dysfunction and lymphopenia. In addition, it has been reported that immunosuppressive drugs such as interleukin antagonists and steroids used in the treatment of severe COVID-19 may increase the risk of CAPA.^9^-^10^

Mucormycosis is a rare, life-threatening opportunistic fungal infection caused by Mucorales. Diabetes, systemic corticosteroids, neutropenia and immunosuppression are conditions known to increase the risk of mucormycosis.^11^ A series of COVID-19 secondary mucormycosis has been reported and defined as COVID-19-related mucormycosis (CAM).^12^ Alveolar damage and immune dysfunction observed in COVID-19 patients may cause Rhizopus spp. to invade lung tissue and Aspergillus spp. 13 The aim of this study was to define the risk factors for IMI related with severe COVID-19 pneumonia.

## Materials and methods

### Study Design and Patients

This case-control study was carried out in a tertiary hospital with 1607 beds and 253 intensive care beds prospectively. Patients who were followed up in the intensive care unit due to severe COVID-19 between August 2020 and June 2021 were included in this study. Patients with secondary IMI were determined as the study group. The control group was randomly selected from severe COVID-19 patients who were treated in the intensive care unit during the same period and had no secondary infection (bacterial or fungal). The patient who was admitted due to COVID-19 right after the patient diagnosed with IMI was included.

### Definitions

COVID-19 pneumonia; thoracic tomography (CT) positivity in upper respiratory tract samples suitable for CO-RADS 4, CO-RADS 5 and CO-RADS 6 categories where typical uptake for COVID-19 is expressed and SARS-CoV-2 RT PCR test is positively identified as patients.^14^ Severe COVID-19 pneumonia was defined as fever and respiratory tract infection findings and the presence of one of the following: respiratory rate >30/min, defined as severe respiratory distress (dyspnea, use of extra respiratory muscles), presence of oxygen saturation <90% in room air (PaO2/FiO2 < 300 in the patient receiving oxygen).^15^ CAPA classification (proven/probable) was performed by using the 2020 ECMM/ISHAM consensus criteria, using a combination of microbiology, imaging and clinical factors.^9^

The diagnosis of mucormycosis was determined according to the guidelines for the clinical managements of mucormycosis published by the European Confederation of Medical Mycology in collaboration with the Mycoses Working Group Education and Research Consortium.^16^ Institutional protocol for treating COVID-19 patients; All patients were treated with favipiravir (1600 mg loading dose and 800 mg/day maintenance dose, orally). Supportive therapy consisted of oxygen and fluid supplements, and also vasopressor agents if necessary. Steroids and high-dose steroids were used in accordance with the national guidelines for patients with increased inflammatory markers.^17^ In patients who needed oxygen therapy support due to respiratory distress, 6mg/day dexamethasone or 1mg/kg methylprednisolone was used. Pulse steroid (≥250 mg/day methyl prednisolone for up to 3 days) was given in patients with increased oxygen demand or acute phase response within 24 hours despite treatment. Tocilizumab was administered with the low-dose protocol developed by our institution in order to reduce side effects (After administration of 80 or 100 mg, a further 80 or 100 mg repeat dose was administered within 24-48 hours).^18^ Anakinra was used in accordance with the national guideline recommendation (ranging from 100 mg subcutaneous injection once or twice a day to 200 mg IV administration 3 times a day in the presence of severe symptoms, depending on the severity of the patient's clinical findings).^17^

### Mold Identification and Antifungal Tests

Tissue samples were taken by surgical debridement from patients with a preliminary diagnosis of rhinoorbital mucormycosis. Aseptate hyphae are shown in KOH assembly and tissue-prepared staining (Figure 1). Tissue samples were incubated in two Sabouraud dextrose agar (SDA; Oxoid, UK) for 7 days at 25 °C and 37°C. The colonial appearance was cottony and velvety (Figure 2). Bronchoscopic/nonbronchoscopic lavage fluid samples were obtained from patients with a pre-diagnosis of CAPA. The sample was treated with KOH and examined under direct microscopic examination. A bronchoalveolar specimen was seeded on Sabouraud Dextrose Agar (SDA, Oxoid, UK) with or without antibiotics and incubated at 37°C and 25°C. At 25°C, SDA colonies were initially white and quickly turned black with conidial production after one week of incubation (Figure 3). In microscopic examination, dark or dark brown spores were observed in septal hyphae and conidial heads (two rows). DNA analysis of clinical strains was carried out in a private laboratory (EurX GeneMATRIX Plant & Fungi DNA).^18^ Identification of the fungal complex using polymerase chain reaction (PCR) products, primers and Sequencer (Applied Biosystems, Foster City, CA) was performed based on finally sequencing the PCR amplicons of the internally replicated spacer 1 and internally replicated spacer 2.^19^ Sequence analysis data were analyzed using the “National Center for Biotechnology Information (Bethesda, USA)” BLAST system (http://www.ncbi.nlm.nih.gov/BLAST/).

Susceptibility testing was performed using the E-test (bioMerieux (France). Aspergillus spp. was obtained from 7-day-old cultures on potato dextrose agar. One milliliter of sterile normal saline was added to the cultures and mixed gently with a Pasteur pipette to suspend the conidia. Sterile NaCl with 0.5 McFarland standard or an optical density of 0.1-0.12. Seeding of RPMI-2 glucose test medium at 530 nm (corresponding to approximately 106 colony forming units/mL) was performed as described above and allowed to dry on the surface of the medium. Etest® strips were gently placed on agar plates were incubated for 24-48 or 72 hours if needed at 35 °C MICs against voriconazole, caspofungin and amphotericin B were determined for all Aspergillus spp.

Serum galactomannan was determined for use in the Platelia Aspergillus EIA assay (Bio-Rad Laboratories, Marnes, France) according to the manufacturer's instructions. GM value ≥0.5 ng/mL was accepted as a positive result.

### Statistical analysis

Statistical analysis was performed using the SPSS 22.0 (IBM Corp., Armonk, NY, USA) package program. The Shapiro-Wilk test was performed to check the normality assumption of the data. Categorical variables were expressed as numbers and percentages, and Chi-square or Fisher's Exact Test analysis was used for comparisons. Variables that P-value ≤0.05 were included in the multivariate logistic regression analysis. P-value ≤0.05 was considered statistically significant in all analyzes.

### Ethical approval

This study was approved by the clinical research ethics committee of xxx Hospital (Date: 10.12.2020 Number: 226).

## Results

### Patient characteristics

A total of 39 patients, including 20 patients in the IMI group and 19 patients in the control group, were included into the study. The clinical and demographic characteristics of the patients are given in Table 1. Twenty-seven (69.2%) of the patients were male, and the median age was 67 (23-89). Between IMI and control group; there was no difference in terms of age and gender (p=0.050 and p=0.501), comorbidities present in the patients were similar in both groups (p>0.05), the rates of mild ARDS, moderate ARDS, and severe ARDS were not statistically different between the ICE group and the control group (p=0.333, p=0.751, p=0.661). APACHE II scores were similar (0.945) While the need for standard O2 and high-flow O2 support did not differ between the groups (p=0.527, p=0.514), three patients (15.8%) in the control group had a history of intubation (p=0.106). The 28-day mortality was higher in the IMI group (60.0% vs. 15.8%, p=0.005).

**Table 1:** Demographic and Clinical Characteristics of the Patients

	Invasive mold infection	Control	Total	P	Multivariate Analysis	
	(n=20)(%)	(n=19) (%)	(n=39)(%)		OR (95% CI)	*P*
Age-median (min-max)	73 (23-89)	62 (47-86)	67 (23-89)	0.050		
Male gender	15 (75.0)	12 (63.2)	27 (69.2)	0.501		
**Symptoms of COVID-19**						
Fever	9 (45.0)	10 (52.6)	19 (48.7)	0.752		
Cough	16 (80.0)	14 (73.7)	30 (76.9)	0.716		
Respiratory Distress	17 (85.0)	13 (68.4)	30 (76.9)	0.273		
**Comorbidities**						
At least a comorbidity	16 (80.0)	13 (68.4)	29 (74.4)	0.480		
Diabetes mellitus	11 (55.0)	8 (42.1)	19 (48.7)	0.527		
Hypertension	10 (50.0)	10 (52.6)	20 (51.3)	1.000		
COPD	2 (10.0)	3 (15.8)	5 (12.8)	0.661		
Coronary arter disease	2 (10.0)	3 (15.8)	5 (12.8)	0.661		
**ARDS Severity (due to COVID-19)**						
Mild	6 (30.0)	9 (47.4)	15 (38.5)	0.333		
Moderate	10 (50.0)	8 (42.1)	18 (46.2)	0.751		
Severe	4 (20.0)	2 (10.5)	6 (15.4)	0.661		
**Respiratory support**						
Standart O2	11 (55.0)	8 (42.1)	19 (48.7)	0.527		
High-flow O2	6 (30.0)	8 (42.1)	14 (35.9)	0.514		
Non-Invasive mechanical ventilation	3 (15.0)	-	3 (7.7)	0.231		
Invasive mechanical ventilation	-	3 (15.8)	3 (7.7)	0.106		
**Immunosuppressive Therapy**						
**Steroid**	18 (90.0)	3 (15.8)	21 (53.8)	<0.001	25.712 (2.257-292.909) 0.009	
Dexamethasone	9 (45.0)	1 (5.3)	10 (25.6)	0.005	2.366(0.165-34.010) 0.527	
Methylprednisolone	9 (45.0)	2 (10.5)	11 (28.2)	0.017	0.423 (0.029-6.075) 0.527	
**Immunomodulatory agent**	8 (40.0)	1 (5.3)	9 (23.1)	0.010	1.984 (0.137-28.820) 0.616	
Tocilizumab	5 (25.0)	1 (5.3)	6 (15.4)	0.182		
Anakinra	3 (15.0)	-	3 (7.7)	0.231		
**Prognosis**						
Mortality up to 28^th^ day	12 (60.0)		15 (38.5)	0.008		
		3 (15.8)				

In the IMI group, 18 (90.0%) patients were treated with steroids, whereas three (15.8%) patients in the control group were treated with steroid (p=<0.001). Steroid dose and duration were similar in IFI and control groups. For the treatment of COVID-19, dexamethasone in nine patients (45.0%) with IMI and one (%5.3) control patient (p=0.005); Methylprednisolone was used in nine patients (45.0%) in the IMI group and in two (10.5%) patients in the control group (p=0.017). In addition, immunomodulatory agents (tocilizumab or anakinra) were used in eight patients (40.0%) with IMI and one patient (5.3%) in the control group (p=0.010) (Table 1).

Invasive aspergillosis was observed in a total of 13 patients (65%). Of these patients, 12 (60.0%) have CAPA and one (5.0%) acute invasive fungal sinusitis due to Aspergillus spp. Seven patients (35.0%) developed CAM (Table 2).

**Table 2:** Risk factors, clinics and agents of IMI patients

	Respiratory support	High-dose steroid (drug/dose)	Steroid (drug/dose)	Immunomodulator Agent (Drug/totally dose)	Invasive mold infection	Fungal Agent	Outcome
**Case 1**	NIMV		Dexamethasone 16 mg/day	Anakinra/800 mg	CAPA	Aspergillus fumigatus	Death
**Case 2**	Standard O2		Dexamethasone 16 mg/day		CAPA	Aspergillus Flavus	Death
**Case 3**	Standard O2				CAPA	Aspergillus fumigatus	Alive
**Case 4**	NIMV		Dexamethasone 8 mg/day		CAPA	Aspergillus niger	Death
**Case 5**	Standard O2		Dexamethasone 6 mg/day	Tocilizumab/100 mg	Rhino-orbital mucormycosis	Cladosporium allicinum	Alive
**Case 6**	Standard O2		Dexamethasone 8 mg/day		Rhino-orbital mucormycosis	Rhizopus Oryzae	Alive
**Case 7**	Standard O2		Dexamethasone 16 mg/day		Rhino-orbital mucormycosis	Rhizopus Oryzae	Death
**Case 8**	Standard O2		Dexamethasone 16 mg/day		Rhino-orbital mucormycosis	Rhizopus Oryzae	Death
**Case 9**	Standard O2				CAPA	Aspergillus niger	Alive
**Case 10**	HFNO	Methylprednisolone 1000 mg			Rhino-orbital mucormycosis	Rhizopus Oryzae	Death
**Case 11**	Standard O2		Methylprednisolone 60 mg/day	Anakinra/800 mg	CAPA	Aspergillus fumigatus	Death
**Case 12**	Standard O2		Dexamethasone 16 mg/day	Tocilizumab 100 mg	Invasive fungal sinusitis	Lichtheimia Corymbifera/Aspergillus terreus	Alive
**Case 13**	HFNO	Methylprednisolone 750 mg		Tocilizumab 200 mg	CAPA	Aspergillus fumigatus	Death
**Case 14**	HFNO		Methylprednisolone 40 mg/day	Tocilizumab 200 mg	CAPA	Aspergillus terreus	Alive
**Case 15**	NIMV		Methylprednisolone 60 mg/day		CAPA	Aspergillus fumigatus	Death
**Case 16**	HFNO		Methylprednisolone 40 mg/day	Tocilizumab 200 mg	CAPA	Aspergillus fumigatus	Alive
**Case 17**	Standard O2		Methylprednisolone 40 mg/day	Anakinra/800 mg	CAPA	Aspergillus fumigatus	Alive
**Case 18**	HFNO		Methylprednisolone 80 mg/day		CAPA	Aspergillus fumigatus	Death
**Case 19**	HFNO	Methylprednisolone 750 mg			Invasive fungal sinusitis	Aspergillus flavus	Death
**Case 20**	Standard O2		Methylprednisolone 40 mg/day		Rhino-orbital mucormycosis	Rhizopus oryzae	Death

### Isolates

A total of 21 agents were isolated in 20 patients who were followed up with IMI (Table 2). Aspergillus fumigatus was the causative agent in eight (38.1%) patients and Rhizopus oryzae in five (23.8%) patients. Aspergillus terreus and Lichtheimia corymbifera were isolated together in the 12th case diagnosed with acute invasive fungal sinusitis. Antifungal susceptibility tests and galactomannan results are presented in Table 3. Proven CAPA was detected in two patients who had bronchoscopic lavage. Galactomannan test was studied in three patients and it was positive (Table 3).

**Table 3:** Fungal agents of IMI patients

			Susceptibility			Galactomannan	CAPA
			Minimum Inhibitor concentration (MIC) (µg/mL)			(ng/mL)	
	Fungal Agent	Sequence analysis	Voriconazole	Amphotericin B	Caspofungin		
**Case 1**	Aspergillus fumigatus	JQ776545.1	1.00	4.00	2.00		Probable
**Case 2**	Aspergillus Flavus	MT645322.1	1.00	2.00	1.50		Probable
**Case 3**	Aspergillus fumigatus	MT597427.1	0.38	2.00	0.25	25.0	Proven
**Case 4**	Aspergillus niger	MT620753.1	0.25	1.00	1.50		Probable
**Case 5**	Cladosporium allicinum	MF472917.1					
**Case 6**	Rhizopus Oryzae	MT540020.1					
**Case 7**	Rhizopus Oryzae	MT316366.1					
**Case 8**	Rhizopus Oryzae	MH715977.1					
**Case 9**	Aspergillus niger	MT597435.1	0.25	1.00	0.19		Proven
**Case 10**	Rhizopus Oryzae	MT603963.1					
**Case 11**	Aspergillus fumigatus	MT635279.1	1.00	4.00	2.00		Probable
**Case 12**	Lichtheimia Corymbifera/	MT316349.1/					
	Aspergillus terreus						
		MT558939.1					
**Case 13**	Aspergillus fumigatus	MT635279.1	1.00	4.00	2.00	2.0	Probable
**Case 14**	Aspergillus terreus	MT558939.1	0.50	3.00	0.75		Probable
**Case 15**	Aspergillus fumigatus	MT597427.1	0.50	1.00	1.50		Probable
**Case 16**	Aspergillus fumigatus	MT597427.1	0.38	1.00	1.50	4.0	Probable
**Case 17**	Aspergillus fumigatus	MN634608.1	0.50	12.00	0.38		Probable
**Case 18**	Aspergillus fumigatus	MH864623.1	0.50	<32.00	0.50		Probable
**Case 19**	Aspergillus flavus	MT645322.1	1.00	2.00	1.50		Probable
**Case 20**	Rhizopus oryzae	LC514313.1					

### Risk factors for IMI

In multivariate analysis, steroid use was identified as the most important risk factor for the development of IMI (90.0% vs. 15.8%, OR: 25.712, p=0.009).

## Discussion

In this study, first of all, the clinical characteristics and risk factors of patients who were followed in the intensive care unit due to COVID-19 and developed IMI were evaluated. The age and demographic data of the patients with and without IMI were similar. Steroid use for treating COVID-19 was statistically significantly higher in the IMI group. The 28-day mortality was higher in the IMI group (60.0% vs. 15.8%).

The diagnosis and treatment of IMI is very difficult. It has been reported in the literature that the incidence of IMI associated with COVID-19 has increased dramatically in recent times.^20^,^21^

In a multicenter study evaluating risk factors for mucormycosis associated with COVID-19 in India, steroid use was cited as a risk factor. Especially in patients without hypoxemia, a steroid used when not indicated has been associated with CAM development.^22^ Also in our study, steroid use was the most critical risk factor for IMI. This may be since steroids increase the blood glucose level and lymphopenia.

It is shown that, pro-inflammatory cytokines such as IL-1, IL-6, IL-18 activated in COVID-19 related MAS.^23^,^24^ In patients with cytokine storm or MAS, immunosuppressive drugs are recommended to prevent tissue damage caused by the increased autoimmune response. Tocilizumab, one of the interleukin receptor antagonists, is included in the COVID-19 treatment guidelines.^5^ It has been reported that development of IMI increased due to the increase in the use of these drugs. In a multicenter study published by the European Confederation of Medical Mycology, tocilizumab was identified as a risk factor to the development of CAPA.^10^ In contrast, according to our data, the frequency of tocilizumab or anakinra was similar between IMI patients and the control group. In the practical approach of our center, using less than the recommended doses of tocilizumab in the guidelines may have provided this.

Intubation has been clearly identified as a risk factor for CAPA in literature. In the same multicenter study, any invasive respiratory support was found to be a risk factor for CAPA.^10^ None of our patients who developed IMI had a history of intubation is another noteworthy point of our study. This suggests that epithelial and alveolar damage from SARS-CoV-2 is more efficient in the development of CAPA. It was observed that the majority of patients who had invasive fungal sinusitis and rhino-orbital mucormycosis received standard O2. Nasal O2 administered intranasally with a nasal cannula causes dryness and damage to the nasal and sinus tissue. Considering the increased risk of invasion and proliferation of the Mucorales spp. group in the sinus environment where high oxygen levels are reached, nasal O2 support may be facilitating the development of mucormycosis.

The most important limitation of this study is the small number of patients. The limited number of patients in the subgroups prevented detailed analysis and comparisons. Studies with a larger number of patients will contribute to the literature.
